# Shape-dependent toxicity and mineralization of hydroxyapatite nanoparticles in A7R5 aortic smooth muscle cells

**DOI:** 10.1038/s41598-019-55428-9

**Published:** 2019-12-12

**Authors:** Ling-Hong Huang, Xin-Yuan Sun, Jian-Ming Ouyang

**Affiliations:** Institute of Biomineralization and Lithiasis Research, Jinan University, Guangzhou, 510632 China

**Keywords:** Bioinorganic chemistry, Cell death, Risk factors

## Abstract

Vascular smooth muscle cell damage is a key step in inducing vascular calcification that yields hydroxyapatite (HAP) as a major product. The effect of the shape of HAP on the damage to vascular smooth muscle cells has yet to be investigated. In this study, we compared the differences in toxicity of four various morphological nano-HAP crystals, namely, H-Rod, H-Needle, H-Sphere, and H-Plate, in rat aortic smooth muscle cells (A7R5). The sizes of these crystals were 39 nm × 115 nm, 41 nm ×189 nm, 56 nm × 56 nm, and 91 nm × 192 nm, respectively. Results showed that all HAPs decreased cell viability, disorganized cell morphology, disrupted cell membranes, increased intracellular reactive oxygen species concentration, decreased mitochondrial membrane potential, decreased lysosome integrity, increased alkaline phosphatase activity, and increased intracellular calcium concentration, resulting in cell necrosis. The cytotoxicity of the four kinds of HAP was ranked as follows: H-Plate > H-Sphere > H-Needle > H-Rod. The cytotoxicity of each crystal was positively correlated with the following factors: large specific surface area, high electrical conductivity and low surface charge. HAP accelerated calcium deposits on the A7R5 cell surface and induced the expression of osteogenic proteins, such as BMP-2, Runx2, OCN, and ALP. The crystals with high cytotoxicity caused more calcium deposits on the cell surface, higher expression levels of osteogenic protein, and stronger osteogenic transformation abilities. These findings elucidated the relationship between crystal shape and cytotoxicity and provided theoretical references for decreasing the risks of vascular calcification.

## Introduction

Vascular calcifications (VCs) are actively regulated biological processes associated with hydroxyapatite (HAP) crystallization in the extracellular matrix and in middle and intimal cells of the arterial wall^1^. VCs are highly regulated cell-mediated processes, which possess many similarities to bone formation. The center cells of calcification process are vascular smooth muscle cells (VSMCs)^2^. During calcification process, when enough calcium and phosphorus ions accumulate in the matrix vesicles, it will lead to the deposition of calcium phosphate, which will then be converted into octacalcium phosphate and finally converted into insoluble HAP, and HAP repeats nucleation and crystallization in the same approach and expands the deposition area^3^.

Precipitate complexes formed in biological tissues exhibit distinct polymorphic morphology due to different growth environments and different pathological conditions; that is, they appear round, spherical, needle, rod, and laminated particles^4–7^. Villa-Bellosta *et al*.^6^ found that HAP is the only crystalline phase in the calcium and phosphate deposition of lysed and living cells. Rounded crystallites (5–10 nm) exhibiting a random orientation were existed in lysed cells, while the deposits in living cells were composed of 10 nm thick long fiber crystals embedded in an amorphous matrix. Liu *et al*.^5^ obtained and analyzed pellets isolated from the serum of uremia patients through SEM. The pellets have laminated shapes and crystallized needle-like projections (30–500 nm). EDS analysis has demonstrated that the consist of obtained pellets are similar to those of HAP precursor and indicative of CaP crystals, whereas no detectable particles are found in normal serum.

Fully mineralized vesicles in tissues with atherosclerosis are composed of numerous spherical and needle-shaped mineral deposits^4^. Chiou *et al*.^7^ classified calcific depositions into arc, fragmented or punctuated, nodular, and cystic shapes based on ultrasonographic findings.

Many studies^8–14^ have confirmed that HAP crystals cause damage to VSMCs and induce cell phenotype transformation, which in turn promote vascular calcification. For example, exogenous calcifying nanoparticles, which are nanosized complexes of CaP mineral and proteins, are endocytosed by aortic smooth muscle cells, thereby decreasing cell viability, accumulating apoptotic bodies at mineralization sites, and accelerating vascular calcification^11^. Ewence *et al*.^14^ reported CaP crystals induce cell death in human aortic SMCs depending on their size and composition. However, the effects of the morphological characteristics of HAP crystals on cytotoxicity and vascular calcification have not been reported. The size and morphological characteristics of crystals are two important physical parameters that affect cytotoxicity. Sage *et al*.^12^ cultured mouse aorta vascular smooth muscle cells (MASMCs) with different concentrations of nano-HAP for 24 h and found that crystals stimulate the osteogenic transformation of MASMCs in a concentration-dependent manner. Nahar-Gohad *et al*.^10^ showed that HAP induces the osteogenic transformation of rat aortic smooth muscle cells through CaSR- and bone morphogenetic factor-2 (BMP-2)-mediated pathways, thereby leading to the increased expression of the following osteogenic markers: Runt-related transcription factor 2 (Runx2), alkaline phosphatase (ALP), and osteocalcin (OCN).

The inhibitory mechanisms of diethyl citrate (Et_2_Cit), sodium citrate (Na_3_Cit), and phosphonoformic acid in calcification induced by high Pi in mouse aortic smooth muscle cells (MOVAS) have been investigated^15^. The damage mechanism of nanosized HAP on MOVAS and the inhibitory effects of the anticoagulants Et_2_Cit and Na_3_Cit on injury have been explored^16^. Differences in damage to smooth muscle cells caused by nano-HAP crystals with different sizes and shapes have rarely been reported. In this study, the effects of the differences in the morphological characteristics of nano-HAP on rat aortic smooth muscle cell (A7R5) injury and its phenotypic transformation were investigated to provide a basis for determining the effects of the physicochemical properties of crystals on cellular toxicity and vascular calcification.

## Materials and Methods

### Materials

The following materials were used: rat aortic smooth muscle cells (A7R5; Shanghai Cell Bank, Chinese Academy of Sciences); nanosized HAP (Huizhou Weijing Nano New Material Co., Ltd.); DMEM culture medium (HyClone Biochemical Products Co., Ltd., UT, USA); fetal bovine serum (FBS) (Gibco, USA); cell proliferation assay kit (cell counting kit-8, CCK-8), alkaline phosphatase assay kit, BCIP/NBT ALP color development kit, 5,5′,6,6′-tetrachloro-1,1′,3,3′-tetraethyl-imidacarbocyanine iodide (JC-1), anti-fade fluorescence mounting medium, Fluo-4 AM, lactate dehydrogenase (LDH) kit, 2ʹ,7ʹ-dichloro-fluorescein diacetate (DCFH-DA), hematoxylin and eosin staining kit (HE), and phosphatase and protease inhibitors (Shanghai Beyotime Bio-Tech Co., Ltd., Shanghai, China); acridine orange (AO) dye and BCA protein assay kit (Nanjing Jiancheng Technology Co., Ltd., Nanjing, China); alizarin red and Triton X-100 (Xi’an Hurt Biotechnology Co., Ltd.); and annexin V-FITC/PI cell apoptosis and necrosis double dye kits (Beijing 4A Biotech Co., Ltd.), and Ca-sensing receptor inhibitor (NPS-2143) (MCE, New Jersey, USA).

### Experimental methods

#### Characterization of HAP nanoparticles

The phase composition of the HAP nanoparticles was confirmed through X-ray diffraction (D/MAX2400, Japan) with Cu-Kα radiation and Fourier transform infrared spectrometer (FT-IR) (Equinox 55, Bruker, Germany). Their morphological characteristics and size were observed with an ULTRA 55 field emission scanning electron microscope (Zeiss company, Germany) operated at 30 kV. Zeta potentials and conductivity were detected through dynamic light scattering by using a nano-ZS nanoparticle sizer (Malvern, England) at 25 °C. Nitrogen sorption isotherms were measured at −196 °C by using a Tristar 3000 surface area and porosity analyzer (Micromeritics, American). The specific surface area (S_BET_) of the nanoparticles was determined via the Brunauer–Emmet–Teller method.

#### Preparation of fluorescence-labeled HAP

FITC-labeled HAP was prepared via a two-step reaction^17^. A mixture of 0.05 g of HAP and 5 mL of 3-aminopropyltriethoxysilane in 50 mL of anhydrous ethanol was refluxed with continuous stirring under N_2_ environment for 3 h. Then, 0.025 g of FITC was supplied to the mixture at 74 °C for a reaction time of 6 h. The FITC-labeled HAP was obtained after centrifugation and washing by anhydrous ethanol and deionized water.

#### Cell culture

A7R5 cells were grown in DMEM containing 10% FBS in a 5% CO_2_ incubator at 37 °C^18^. The cells were seeded in culture plates at a density of 1.0 × 10^5^ cells/mL, and divided into two groups: (A) control group cells without additional treatment; (B) HAP injury group: H-Rod, H-Needle, H-Sphere, and H-Plate suspended in serum-free medium with different concentrations were co-cultured with A7R5 cells for 24 h or 14 days.

#### Cell viability detection

In this procedure, 100 µL of A7R5 cell suspension with a concentration of 1.0 × 10^5^ cells/mL was seeded per well in 96-well plates for 24 h. A serum-free medium was used to prepare the HAP solution and avoid the complicated effect of serum on HAP toxicity^19^. The cells were exposed to 50, 100, 200, and 400 μg/mL HAP for 24 h, and each group had five parallel holes. Subsequently, 10 μL of CCK-8 dye was added to each well and incubated at 37 °C for 1.5 h. Absorbance was detected by a microplate reader (Safire2, Tecan, Switzerland) at 450 nm.

#### Lactate dehydrogenase (LDH) release assay

After 24 h of incubation, LDH release was measured in accordance with the LDH kit test instructions. The cell culture supernatant was removed, and 100 μL of LDH assay mixture was added to each hole of the 96-well plate. Absorbance was measured by a microplate reader at 490 nm with a reference wavelength of 620 nm.

#### Cell morphology observation

The cell suspension (1 mL) with a concentration of 1 × 10^5^ cells/mL was seeded in 12-well plates. After the cells were exposed to 200 μg/mL HAP crystals with various shapes for 24 h, the cells were observed under a phase contrast microscope (OlyMPUS, CKX41, Japan) (magnification, 400×).

#### Intracellular reactive oxygen species (ROS) detection assay

Following the cell incubations described as above, the cells were exposed to 200 μg/mL HAP crystals with various shapes for 24 h, the samples were stained with DCFH-DA for 20 min. The cells were then observed under a fluorescence microscope (Leica DMRA2, Germany). The fluorescence intensity was detected by using a microplate reader^20^.

#### Observation and detection of lysosomal integrity

Following the cell incubations described as above, the cells were loaded with 5 µg/mL acridine orange (AO) in DMEM for 15 min and incubated with 200 μg/mL HAP crystals with various shapes for 24 h. The distribution of AO was observed under a fluorescence microscope. The fluorescence intensity was measured with excitation at 485 nm and emission at 530 nm (green cytoplasmic AO) and 620 nm (red lysosomal AO)^16^.

#### Measurement of mitochondrial membrane potential (Δψm)

Following the cell incubations described as above, the cells were incubated with 200 μg/mL HAP crystals with various shapes for 24 h. The samples were stained with JC-1 stain, and 2 × 10^4^ cells were detected through flow cytometry (FACS Aria, BD Corporation, CA, USA).

#### Cell apoptosis and necrosis detection

Following the cell incubations described as above, the cells were incubated with 200 μg/mL HAP crystals with various shapes for 24 h. The samples were stained with 5 µL of Annexin V-FITC for 10 min in dark, and then stained with 5 µL propidium iodide. Finally, 2 × 10^4^ cells were detected through flow cytometry.

#### Observation of calcified nodules through alizarin red staining

The medium of each group was replaced every 2 days and incubated with DMEM containing 1% FBS for 14 days. After the treatments were administered, the cells were fixed with paraformaldehyde for 20 min, incubated with 0.1% alizarin red staining (pH = 4.2) for 0.5 h, washed the cells, and observed under a microscope (magnification, 100×). HAP, when used, was applied from days 0 to 1. Then the extracellular HAPs of the cells were removed and the medium containing 1% FBS were used to culture the cells.

Quantitative analysis: The cells were fixed with 70% ethanol for 1 h, washed the cells, and stained with 0.1% alizarin red solution (pH = 4.2) for 1 h. Then the cells were incubated with PBS for 15 min, washed thrice with PBS, and incubated in 10% (w/v) cetylpyridinium chloride for 30 min. Absorbance was detected through a microplate reader at 562 nm, and the absorbance of the supernatant of a group of simple cells without alizarin red was determined.

#### Intracellular and extracellular distribution of HAP

After the cells were exposed to 200 μg/mL FITC-HAP crystals with various shapes for 6 h, this time was required to complete endocytosis without causing too much damage to the cells. The cell membrane was stained with 300 μL of 10 μM DiI for 15 min, and the cells were fixed with paraformaldehyde. Then DAPI staining solution was added to stain the cells for 5 min. The prepared samples were observed under a confocal microscope (LSM510 Meta Duo Scan, Zeiss, Germany).

#### Quantitative analysis of internalized HAP crystals

After the cells were exposed to 200 μg/mL FITC-HAP crystals with various shapes for 6 h, the supernatant was removed. The cells were treated with 0.4 mL of EDTA (5 mM) for 5 min to remove the bound HAP^21^. Then, the cells were rinsed thrice with cold PBS to completely remove the external soluble HAP, and then detected through flow cytometry. The number of cells analyzed in the flow cytometry experiments was 2 × 10^4^.

#### Detection of intracellular calcium concentration

After 24 h of incubation, the cells were stained with 200 µL of Fluo-4/AM staining, incubated at 37 °C for 30 min, washed thrice with PBS, and detected through flow cytometry. The number of cells analyzed in the flow cytometry experiments was 2 × 10^4^.

#### ALP activity assay

The medium of each group was replaced every 2 days and incubated with 1% DMEM containing 1% FBS for 14 days. After the treatments were administered, the cells were fixed with paraformaldehyde for 20 min and incubated with ALP staining in accordance with the manufacturer’s instructions. The stained cells were observed by phase contrast microscope (magnification, 200×). Blue stain indicated a high ALP activity.

Quantitative analysis: After 14 days of incubation, the ALP activity was assessed in the supernatants by using an ALP assay kit. Protein concentrations were detected by a bicinchoninic acid (BCA) protein assay kit, and the ALP activity was normalized for cellular protein content.

#### Osteogenic protein expression by western blotting assays

Cell lysate was prepared through lysis buffer. Equal amounts of protein were loaded and separated on 12% SDS-PAGE and transferred to a PVDF membrane. The membranes were then incubated with primary antibodies against human BMP-2, Runx2, and OCN overnight and detected with the secondary HRP-conjugated antibody. Immune complexes were visualized using an ECL system. Western blot signal intensities were quantified using AlphaEaseFC (Alpha Innotech, San Leandro, CA, USA). The integrated density values for the test and control bands were obtained and shown as their ratio.

#### Statistical analysis

Experimental data were expressed as mean ± standard deviation ($\bar{x}$ ± SD). Experimental results were analyzed statistically using SPSS 13.0 (SPSS Inc., Chicago, IL, USA). Differences in the means between the experimental groups and the control group were analyzed using one-way ANOVA, followed by Tukey’s post hoc test. p < 0.05 indicated significant differences, p < 0.01 corresponded to extremely significant difference, and p > 0.05 denoted no significant differences.

## Results

### Characterization of HAP crystals with various shapes

#### XRD, FT-IR, and SEM characterization of HAP

**Figure 1:**
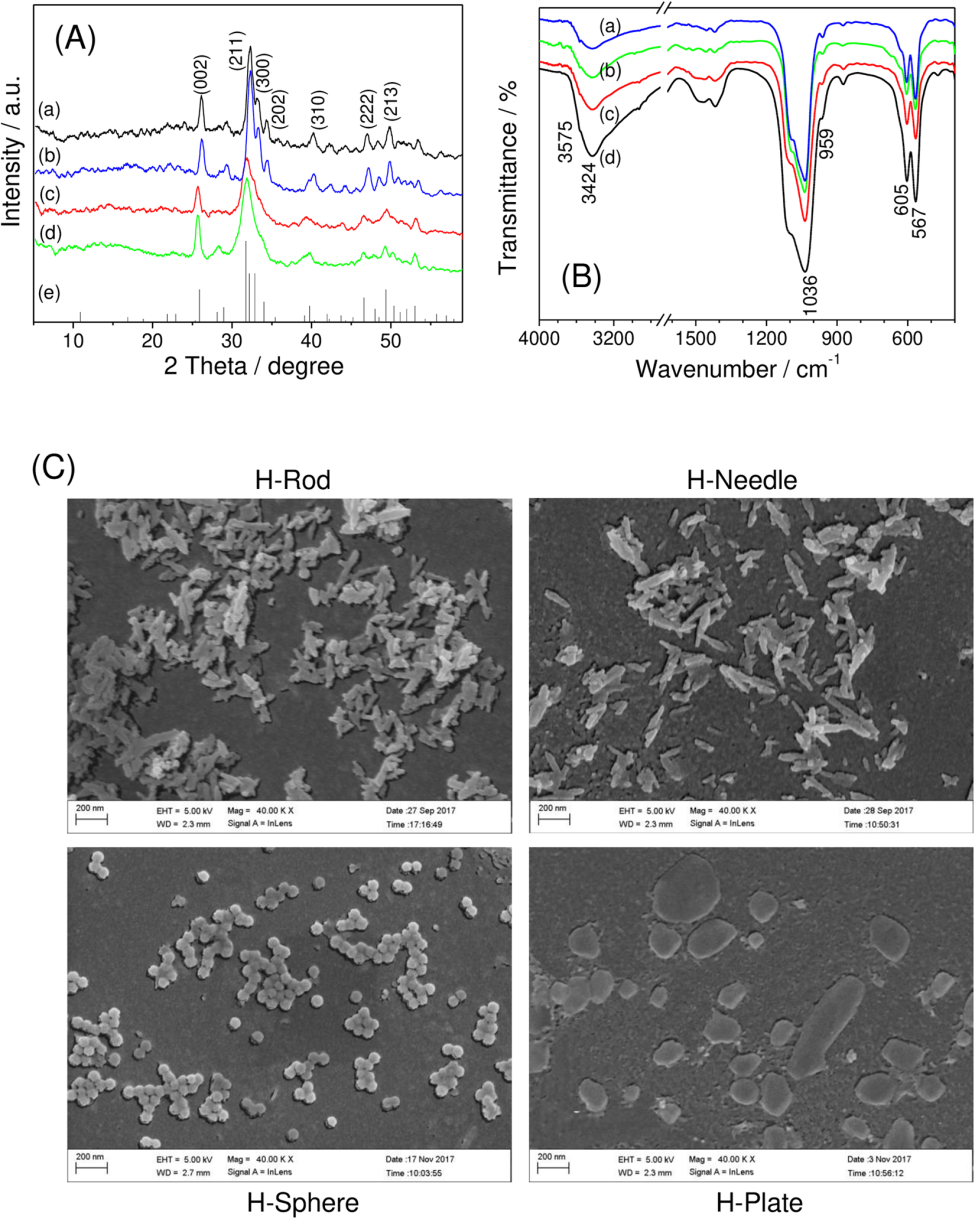
XRD patterns (**A**), FT-IR spectra (**B**), and SEM images (**C**) of HAP crystals with various shapes. (a) H-Rod; (b) H-Needle; (c) H-Sphere; (d) H-Plate; (e) HAP standard spectra (JCPDS no. 09-0432).

Figure 1A shows the XRD patterns of the four different shapes of HAP. The diffraction peaks around 2*θ* = 25.9°, 31.9°, 32.9°, 34.1°, 39.9°, 46.6°, and 49.5° corresponded to the (002), (211), (300), (202), (310), (222), and (213) planes of HAP (JCPDS No. 09-0432), respectively.

Figure 1B illustrates the FT-IR of HAP crystals with various shapes. A broad absorption peak near 3424 cm^−1^ belonged to water adsorbed on the surface of the HAP nanoparticles; the absorption peak near 3575 cm^−1^ belonged to the O–H stretching vibration in HAP; and the vibration peaks at 567, 605, 959, and 1036 cm^−1^ were attributed to the asymmetric stretching vibration peaks of P–O in the PO_4_^3−^ groups^22^.

XRD and FT-IR spectroscopy results showed that all of the four HAP nanoparticles were in their pure phase.

Figure 1C shows the SEM images of the four HAP crystals, namely, rod-like HAP (H-Rod), needle-like HAP (H-Needle), sphere-like HAP (H-Sphere), and plate-like HAP (H-Plate).

#### Zeta potential and conductivity detection of HAP

The zeta potential values of HAP dispersed in pure water and culture medium were negative (Table 1). The absolute value of the zeta potential in the medium obviously reduced because a large number of inorganic ions (e.g., Ca^2+^ and Mg^2+^), amino acids, vitamins, and other auxiliary components are present in DMEM^23–25^. Therefore, the exposed PO_4_^3−^ on the surface of the HAP crystal strongly interacted with Ca^2+^ and Mg^2+^ in the medium, resulting in the partial neutralization of PO_4_^3−^. When PO_4_^3−^ adsorbed a high concentration of cations, the absolute value of the zeta potential decreased.

**Table 1:** Characterization of the physical and chemical properties of HAP with various shapes.

Shape	Crystal length/nm	Crystal width/nm	Zeta in pure water/mV	Zeta in medium/mV	Conductivity in water/mS/cm	Conductivity in medium/mS/cm	Specific surface area S_BET_/m^2^/g
H-Rod	115 ± 23	39 ± 6	−14.5 ± 0.2	−7.02 ± 0.19	0.22 ± 0.025	14 ± 0.058	25.04 ± 2.03
H-Needle	189 ± 27	41 ± 8	−8.74 ± 0.3	−4.58 ± 0.38	0.33 ± 0.003	14.3 ± 0.153	52.46 ± 6.61
H-Sphere	56 ± 9	56 ± 9	−7.00 ± 0.3	−2.57 ± 0.21	0.66 ± 0.008	15.7 ± 0.058	67.03 ± 6.98
H-Plate	192 ± 53	91 ± 31	−17.6 ± 0.5	−8.45 ± 0.33	0.75 ± 0.006	16.1 ± 0.153	185.5 ± 10.23

HAP is a poorly soluble substance, and it is slightly soluble in pure water. The conductivity of HAP in pure water is low (0.22–0.75 mS/cm), whereas the conductivity of the four HAP nanoparticles in the medium is obviously higher (14.0–16.1 mS/cm) than that in pure water possibly because of the large amount of inorganic ions (e.g., Ca^2+^ and Mg^2+^) and amino acids in the medium^23^.

#### *S*_BET_, pore volume, and pore size of HAP

**Figure 2:**
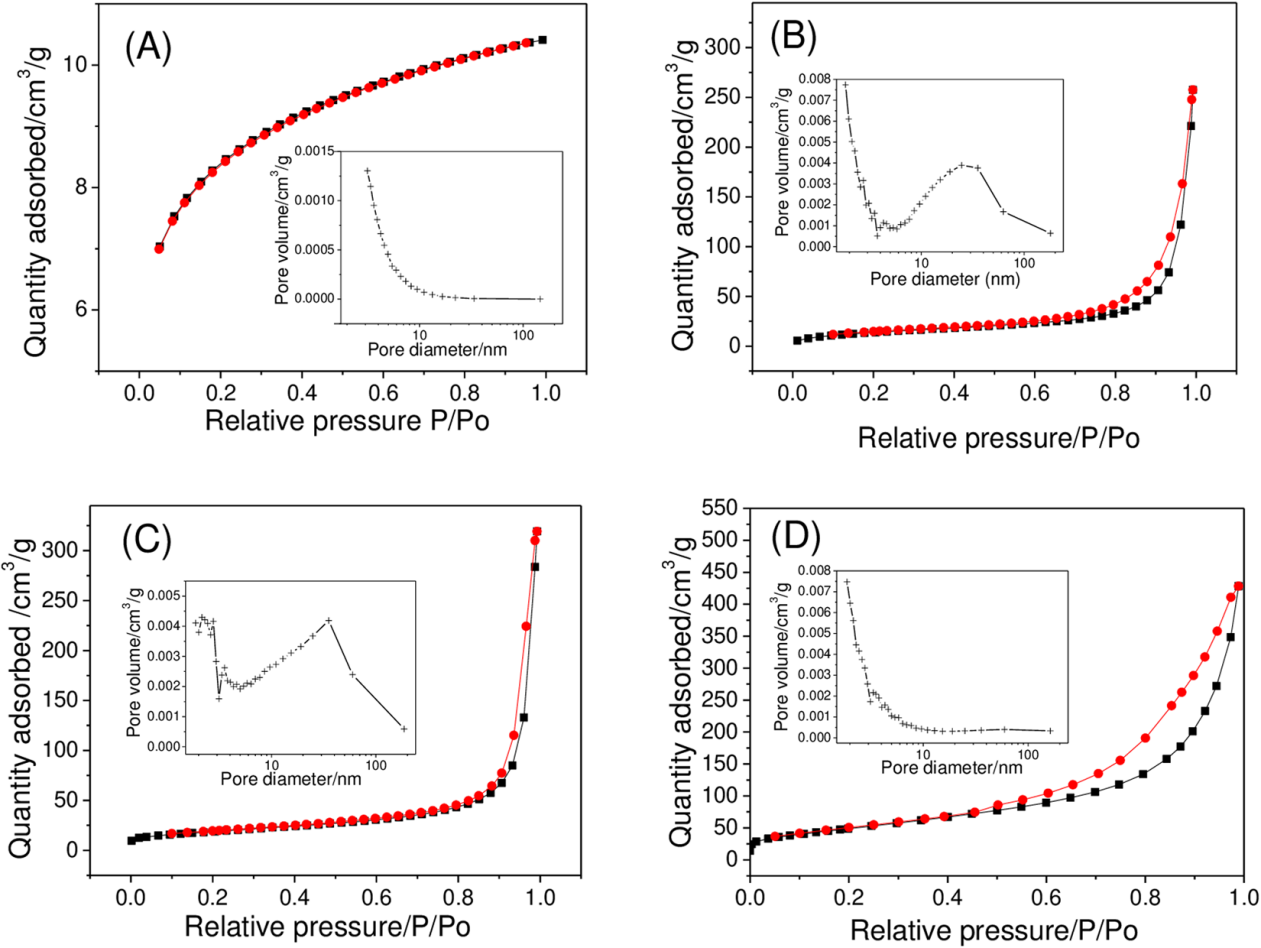
Isothermal nitrogen adsorption desorption curve and pore size distribution of HAP. (**A**) H-Rod; (**B**) H-Needle; (**C**) H-Sphere; (**D**) H-Plate. Red line: adsorption curve; black line: desorption curve.

The adsorption and desorption curves of HAP nanoparticles with various shapes are shown in Fig. 2. The curve of H-Rod is a typical Ι-type (micropore) adsorption isotherm^26^, and its *S*_BET_ and pore size are small (25.04 m^2^/g and 3.14 nm; Table 1). The adsorption and desorption curves of other crystals were a typical type III adsorption isotherm, and the curves tended to be directed to the X axis in the low-pressure section, indicating that the interactions between N_2_ and these crystals were weak, and their surface with holes were rough and had pore sizes of 30.45, 35.29, and 13.44 nm for H-Needle, H-Sphere, and H-Plate, respectively. The *S*_BET_ of the four nanoparticles was ranked in the following order: H-Plate (185.5 m^2^/g) > H-Sphere (67.03 m^2^/g) > H-Needle (52.46 m^2^/g) > H-Rod (25.04 m^2^/g).

### Toxicity of HAP with various shapes on A7R5 cells

**Figure 3:**
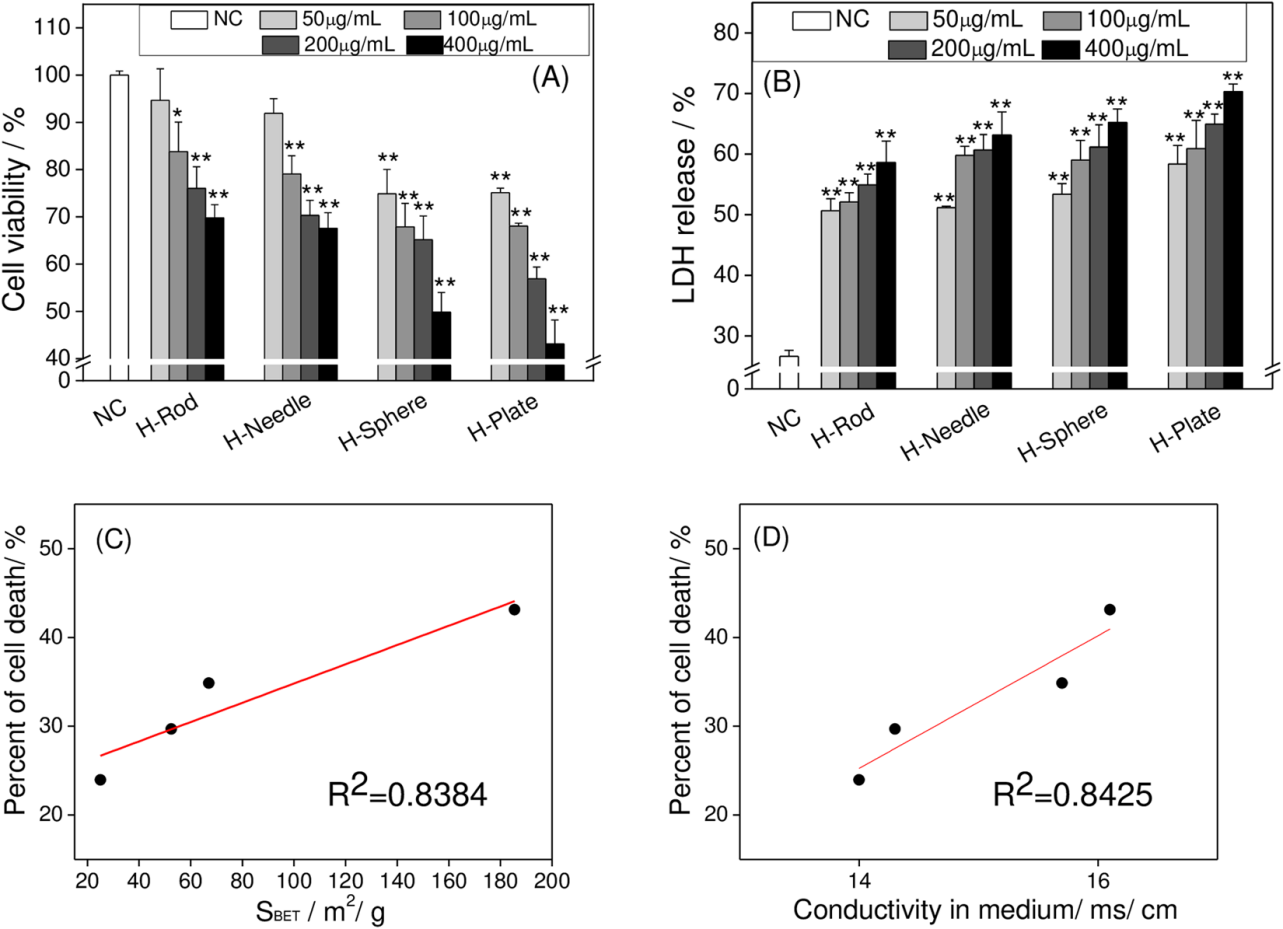
Cell viability (**A**) and LDH release (**B**) assays of A7R5 cells after exposure to different concentrations of HAP crystals with various shapes for 24 h. Correlation curve between specific surface area (**C**), conductivity (**D**) and cytotoxicity. Data were expressed as mean ± SD from three independent experiments. *P < 0.05, **P < 0.01, compared with the control group.

Figure 3A shows the changes in the viability of A7R5 cells treated with four types of HAP for 24 h. The four types of HAP elicited an obvious toxic effect on A7R5 cells in a concentration-dependent manner. The order of cytotoxicity was as follows: H-Plate > H-Sphere > H-Needle > H-Rod. At the crystal concentration of 200 μg/mL, the cell viabilities of H-Plate-, H-Sphere-, H-Needle-, and H-Rod-treated groups were 56.88%, 65.14%, 70.31%, and 76.03%, respectively, which were significantly lower than those of the control group (p < 0.01). The results of the analysis of the correlation of cytotoxicity with *S*_BET_ and electrical conductivity are shown in Fig. 3C,D, respectively. Their correlation coefficients were 0.8384 and 0.8425, respectively. The order of influence of different physical and chemical properties of HAP on its cytotoxicity was as follows: electrical conductivity > *S*_BET_ > Zeta potential.

### Cell membrane damage induced by HAP with various shapes

LDH is a stably present cytosolic enzyme. When the cell membrane is damaged, LDH is released outside the cell, and the increased release of intracellular LDH is considered an important indicator of cell membrane integrity.

In Fig. 3B, all of the four kinds of HAP caused an increase in intracellular LDH release in different degrees and showed a concentration-dependent manner. LDH release was ranked in the following order: H-Plate > H-Sphere > H-Needle > H-Rod. This rule was consistent with cell viability (Fig. 3A).

### Effects of HAP with various shapes on cell morphology

**Figure 4:**
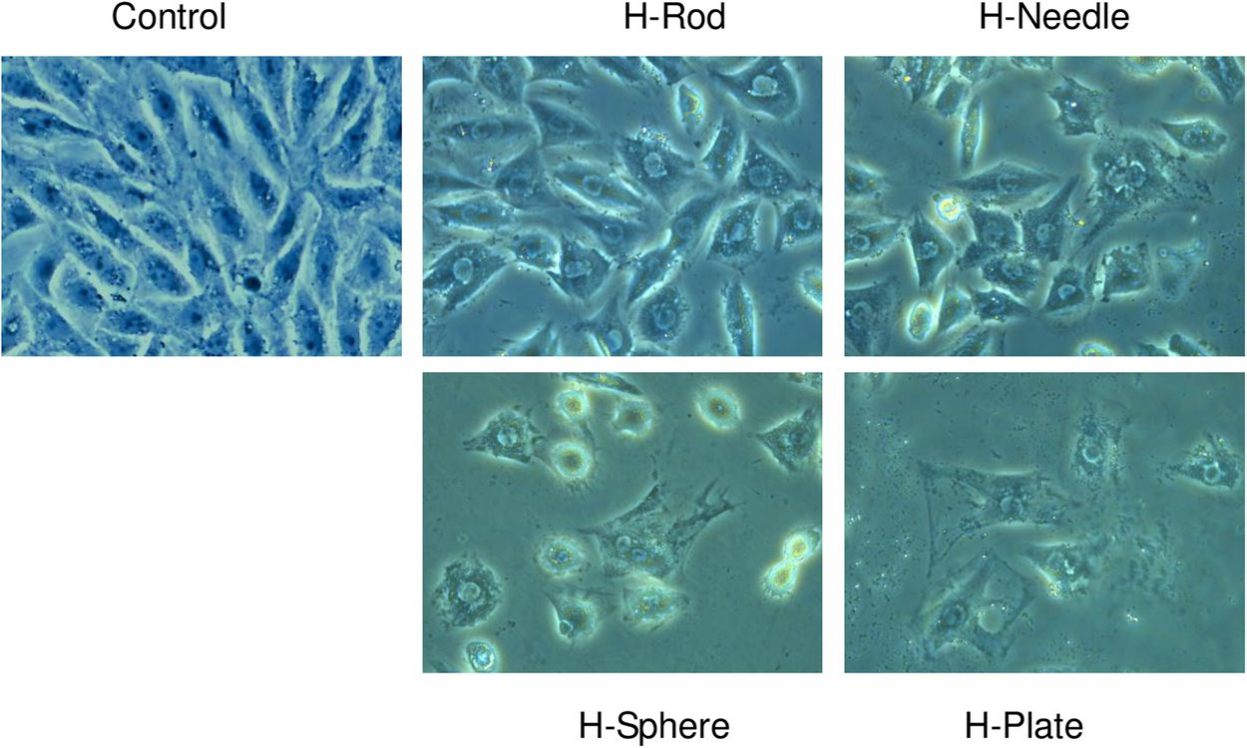
Morphological observation of A7R5 cells exposed to 200 μg/mL HAP crystals with various shapes for 24 h.

Figure 4 shows the morphological changes in A7R5 cells treated with four different morphological HAP nanoparticles. In the control group, the cell showed a plump spindle shape, the morphologies of the cells treated with HAP crystals became disordered. The cell density was obviously reduced. The tight junctions between the cells were destroyed, and some cells had an expanded cytoplasm. The cell damage induced by H-Plate was the most serious. The orders of crystal damage to cell morphology were as follows: H-Plate > H-Sphere > H-Needle > H-Rod.

### Effect of HAP with various shapes on intracellular ROS

**Figure 5:**
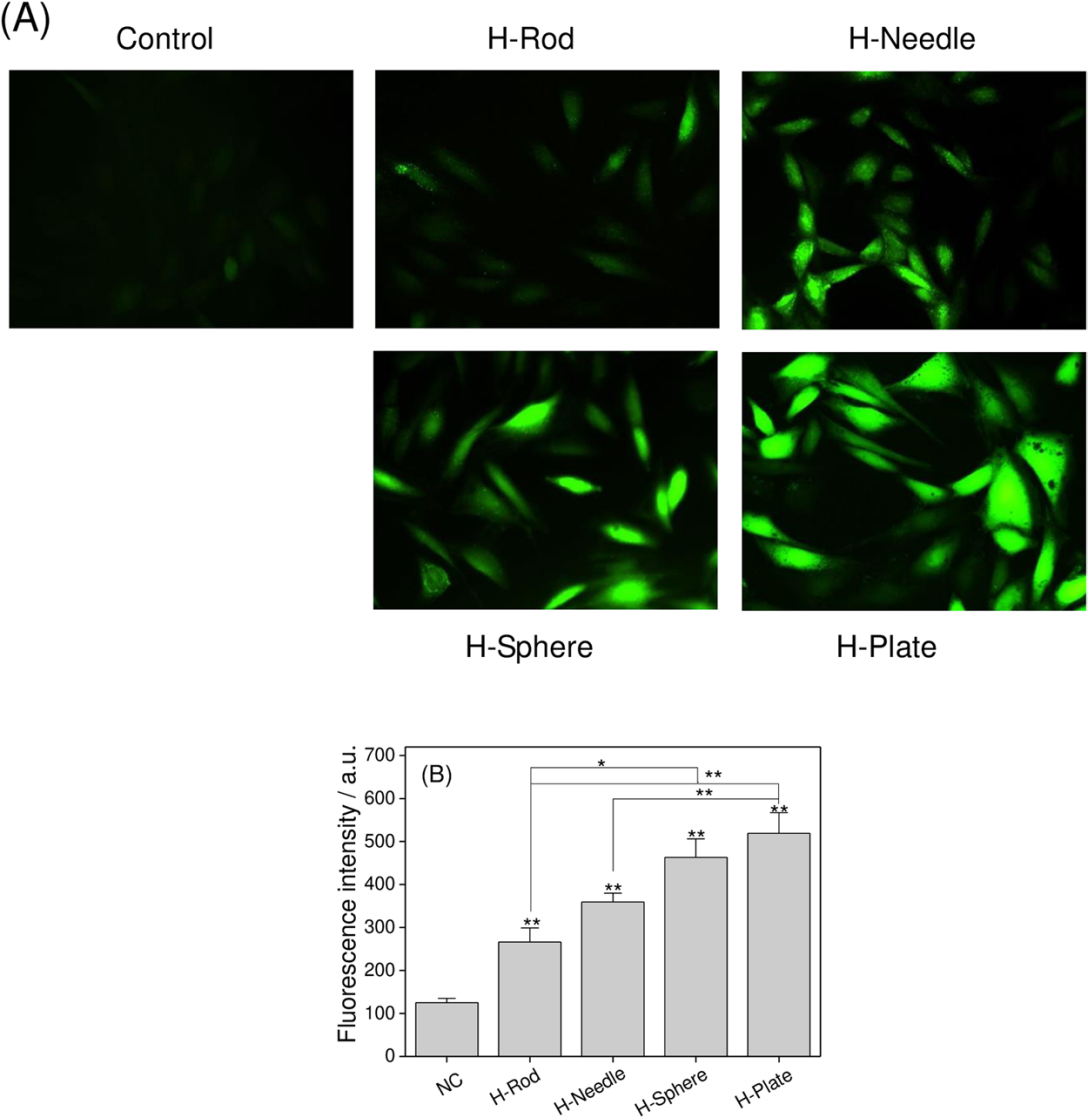
Detection of intracellular ROS level of A7R5 cells exposed to 200 μg/mL HAP crystals with various shapes for 24 h. (**A**) Laser scanning confocal microscope images of intracellular ROS distribution; (**B**) quantitative results of intracellular ROS. The values represent mean ± SD (n = 5), * P < 0.05, **P < 0.01, compared with the control group.

In Fig. 5, the HAP treatment increased ROS generation in A7R5 cells. The cells in the normal group had almost no green fluorescence (Fig. 5A), indicating that the intracellular ROS level was low (125). The green fluorescence of H-Rod-(266), H-Needle-(359), H-Sphere-(463), and H-Plate (519)-treated cells gradually increased, indicating that their intracellular ROS levels gradually increased (Fig. 5B). High cellular ROS levels may lead to apoptosis or necrosis^27^.

### Changes in the lysosome integrity in the cells treated with HAP with various shapes

**Figure 6:**
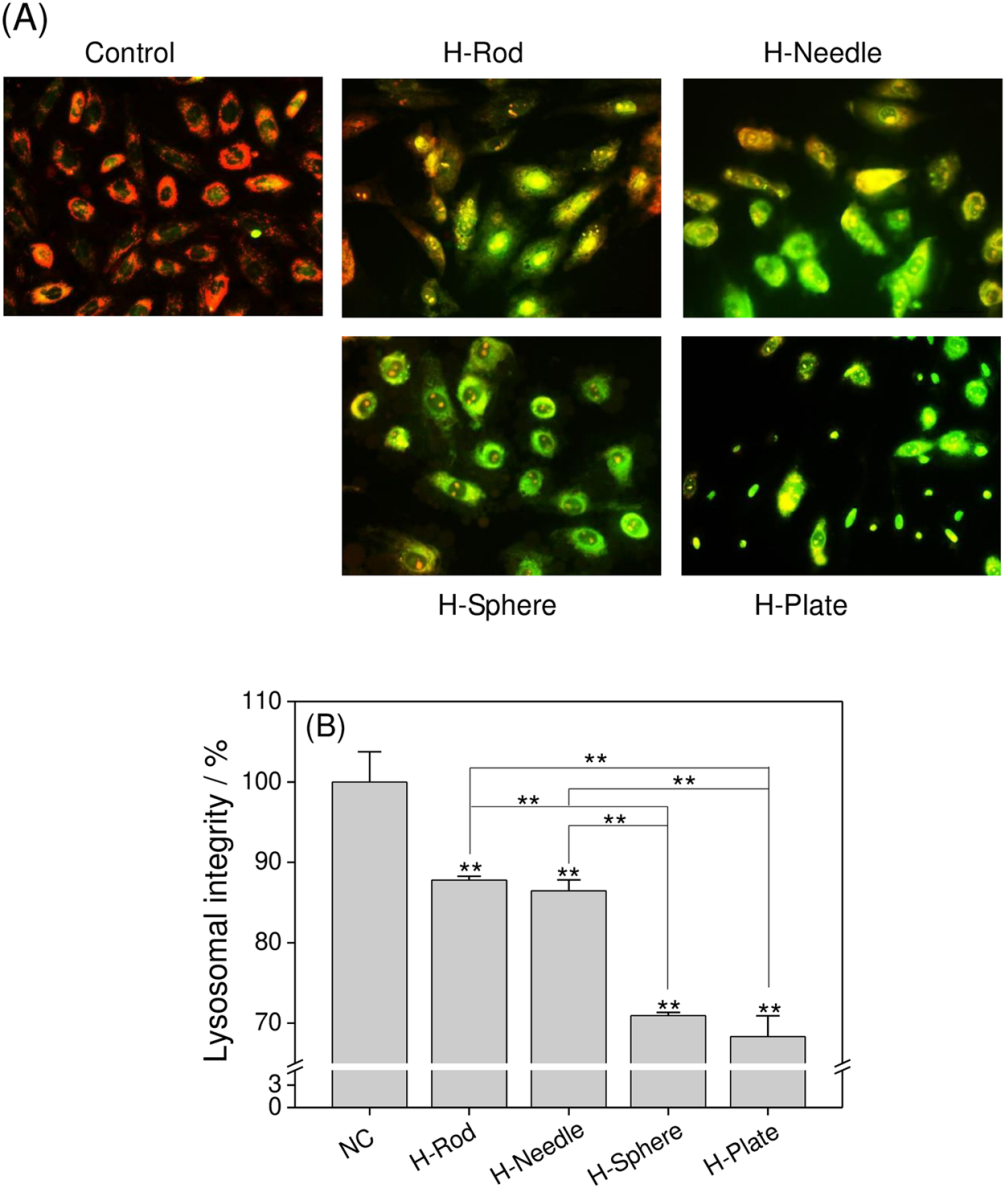
Lysosomal integrity observation of A7R5 cells exposed to 200 μg/mL HAP crystals with various shapes for 24 h. (**A**) Fluorescence microscope observation. (**B**) Quantitative results of lysosomal integrity. The values represent mean ± SD (n = 5), **P < 0.01, compared with the control group.

The degree of lysosomal damage can be determined by Acridine orange (AO) dye^28^. As shown in Fig. 6, the cells in the normal group retained an intact lysosome structure. The emitted red fluorescence of the lysosomes merged with the green fluorescence of the cytoplasm, thereby presenting orange fluorescence. When normal cells were damaged by HAP nanoparticles, the red fluorescence was obviously reduced. The damage to the lysosomes in A7R5 cells treated with the four types of HAP nanoparticles showed the following trend: H-Plate > H-Sphere > H-Needle > H-Rod.

### Effects of HAP with various shapes on Δψm

**Figure 7:**
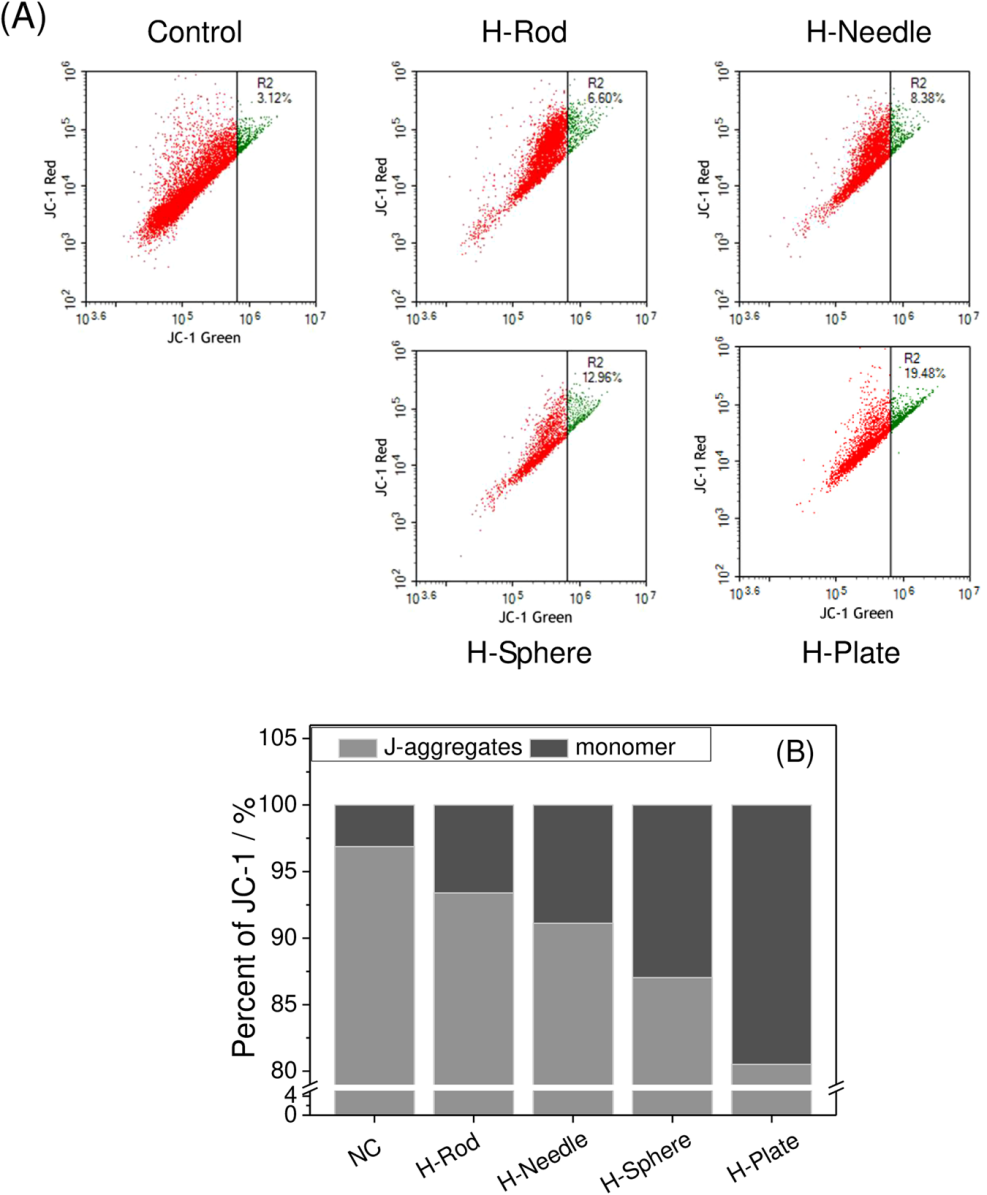
Mitochondrial membrane potential detection (Δ*ψ*m) of A7R5 cells exposed to 200 μg/mL HAP crystals with various shapes for 24 h. (**A**) Flow cytometric data of mitochondrial membrane potential (Δψm). (**B**) quantitative histogram of Δ*ψ*m. The values represent mean ± SD (n = 3).

A decreased Δψm is a hallmark of early cell death. The degree of Δψm can be determined by JC-1 dye^29^. In Fig. 7, the ratio of the cells with low ΔΨm (green fluorescent) to the normal cells was 3.12%, whereas the ratio of the cells in the HAP crystal-damaged group to the cells in the control group obviously increased (6.6%–19.48%), indicating that the different morphologies of HAP caused varying degrees of mitochondrial depolarization. The ratios of low-potential cells in the H-Rod, H-Needle, H-Sphere, and H-Plate groups were 6.6%, 8.38%, 12.96%, and 19.48%, respectively.

### Apoptosis or necrosis induced by HAP with various shapes

**Figure 8:**
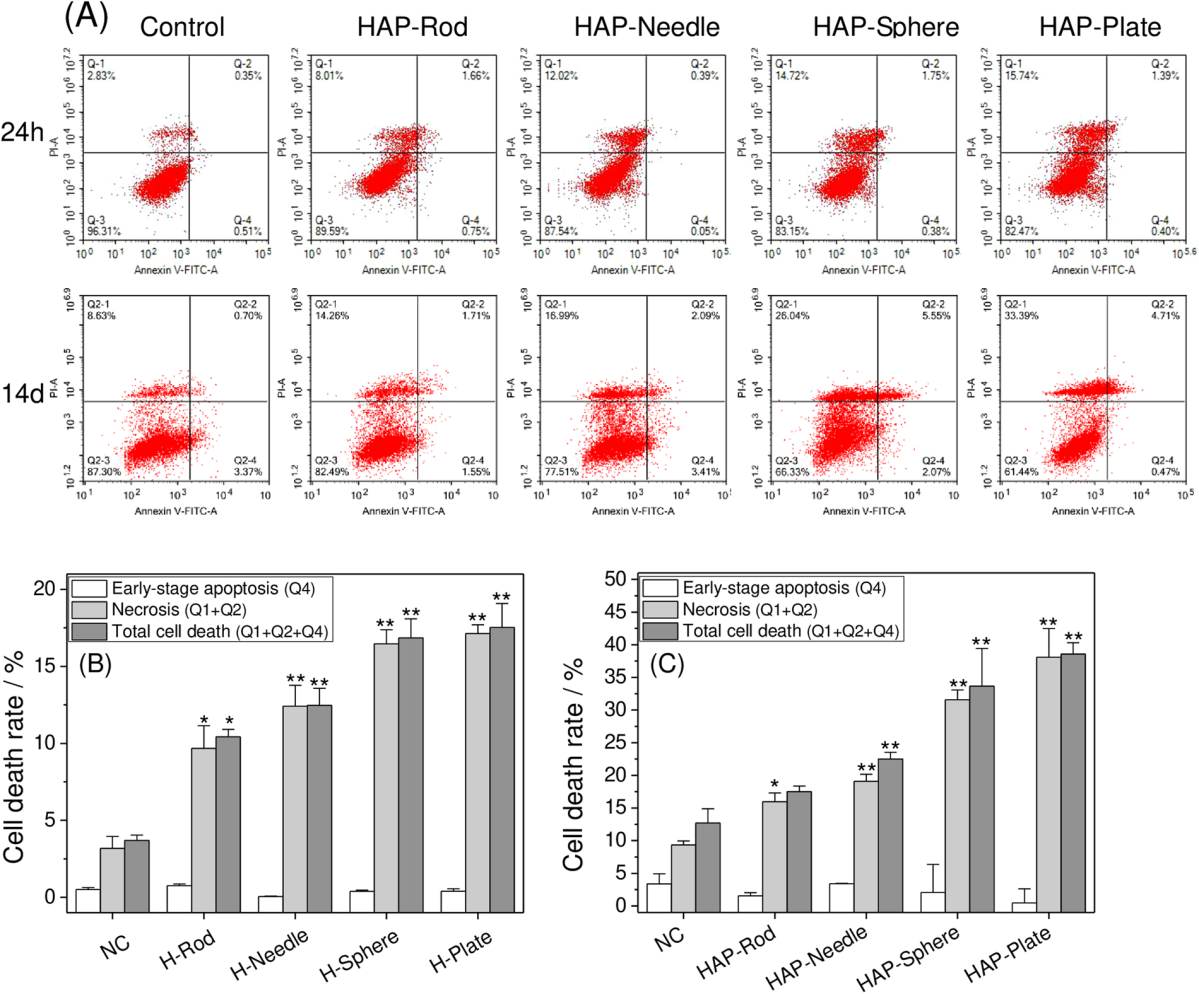
Apoptosis and necrosis assay of A7R5 cells exposed to 200 μg/mL HAP crystals with various shapes for 24 h and 14 d. (**A**) Flow cytometric data of apoptosis and necrosis in A7R5 cells by annexin V/PI double staining. (**B**) Quantitative results of apoptosis and necrosis for 24 h. (C) Quantitative results of apoptosis and necrosis for 14d. Quadrants Q1, Q2, Q3, and Q4 denote the ratio of necrotic cells, necrotic and/or late apoptotic cells, normal cells, and early apoptotic cells, respectively. The values represent mean ± SD (n = 3), *P < 0.05, **P < 0.01, compared with the control group.

Cell apoptosis and necrosis were quantified through Annexin V/PI double staining^30^ (Fig. 8). The cells treated with the four types of HAP exhibited varying degrees of cell necrosis rather than cell apoptosis compared with those of the control group (Fig. 8A,B). The necrosis rates (Q1 + Q2) of the cells treated with H-Plate, H-Sphere, H-Needle, and H-Rod for 24 h were 17.13%, 16.47%, 12.41%, and 9.67%, which respectively increased to 38.1%, 31.59%, 19.08%, and 15.97% when the treatment time was extended to 14 days. The degree of cell necrosis induced by H-Plate and H-Sphere was greater than that induced by H-Rod and H-Needle.

### Calcium depositions on A7R5 cells treated with HAP with various shapes

**Figure 9:**
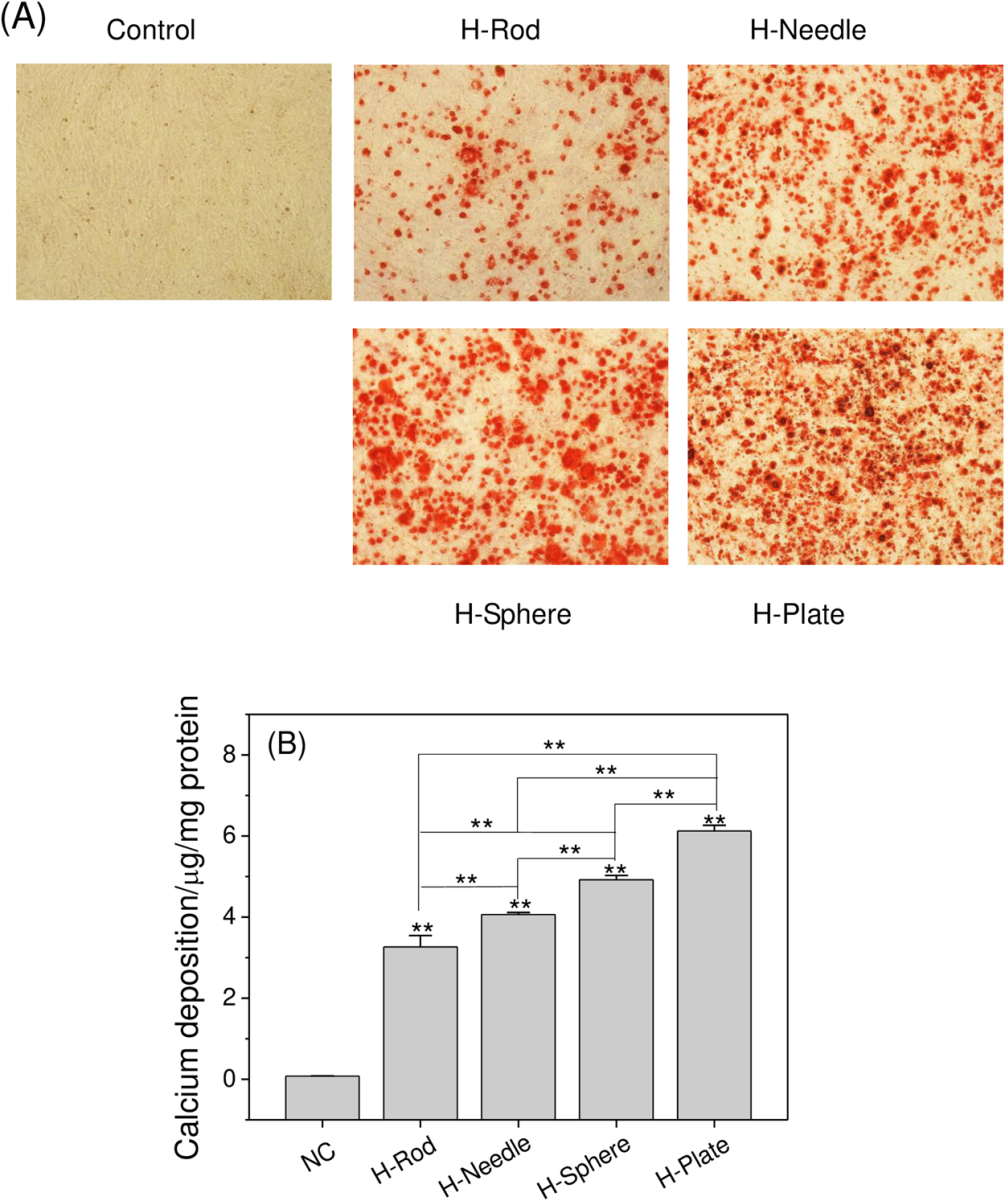
Calcium deposition of A7R5 cell exposed to 200 μg/mL HAP crystals with various shapes for 14 d. (**A**) Microscope observation. (**B**) Quantitative results of calcium deposition. The values represent mean ± SD (n = 5), ** P < 0.01, compared with the control group.

Alizarin red chelates with calcium to form orange-red calcium deposits^31^. Vascular smooth muscle cell injury is a key step in inducing vascular calcification^32^. In Fig. 9, no obvious calcium depositions in normal cells were observed, but the four kinds of HAP caused different degrees of calcium deposition. The changes in the calcium deposition contents were described as follows: H-Plate > H-Sphere > H-Needle > H-Rod.

### HAP distribution inside and outside A7R5 cells

**Figure 10:**
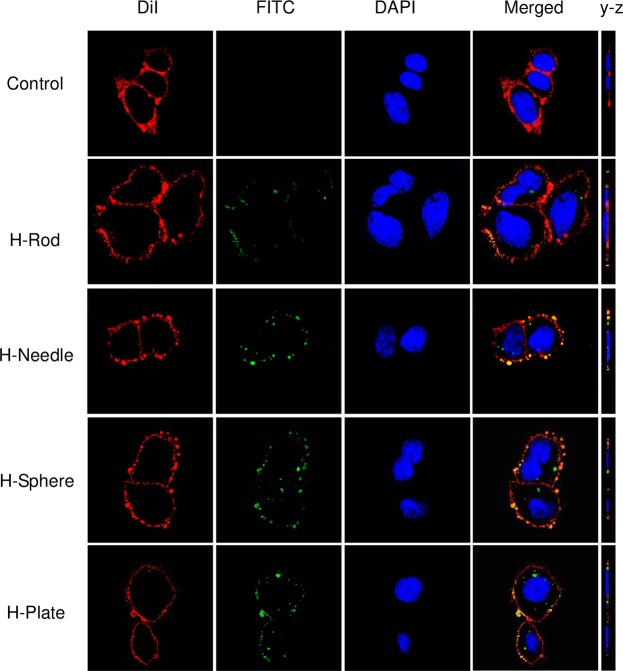
HAP distribution inside and outside A7R5 cells were imaged by a laser scanning confocal microscope to obtain both top view or horizontal (x–y) section and sagittal (y–z) view. A7R5 cells were cultured in the presence of 200 μg/mL of HAP crystals with various shapes for 6 h.

HAP crystals were labeled with FITC (green fluorescence) to verify HAP distribution inside and outside A7R5 cells^33^. The control cells exhibited a typical spindle shape with a full and intact morphology, whereas the HAP crystals caused morphological disorder of cells. A7R5 cells internalized all of the crystals that adhered to the membrane (Fig. 10). The green crystals appeared yellow after they adhered to the red cell membrane. A large yellow area corresponded to the presence of numerous crystals. HAP can encapsulate in cells by vesicles^34,35^. In our study, the internalization degree was higher in H-Sphere and H-Plate than in H-Rod and H-Needle.

### Internalization degree of HAP nanoparticles with various shapes

**Figure 11:**
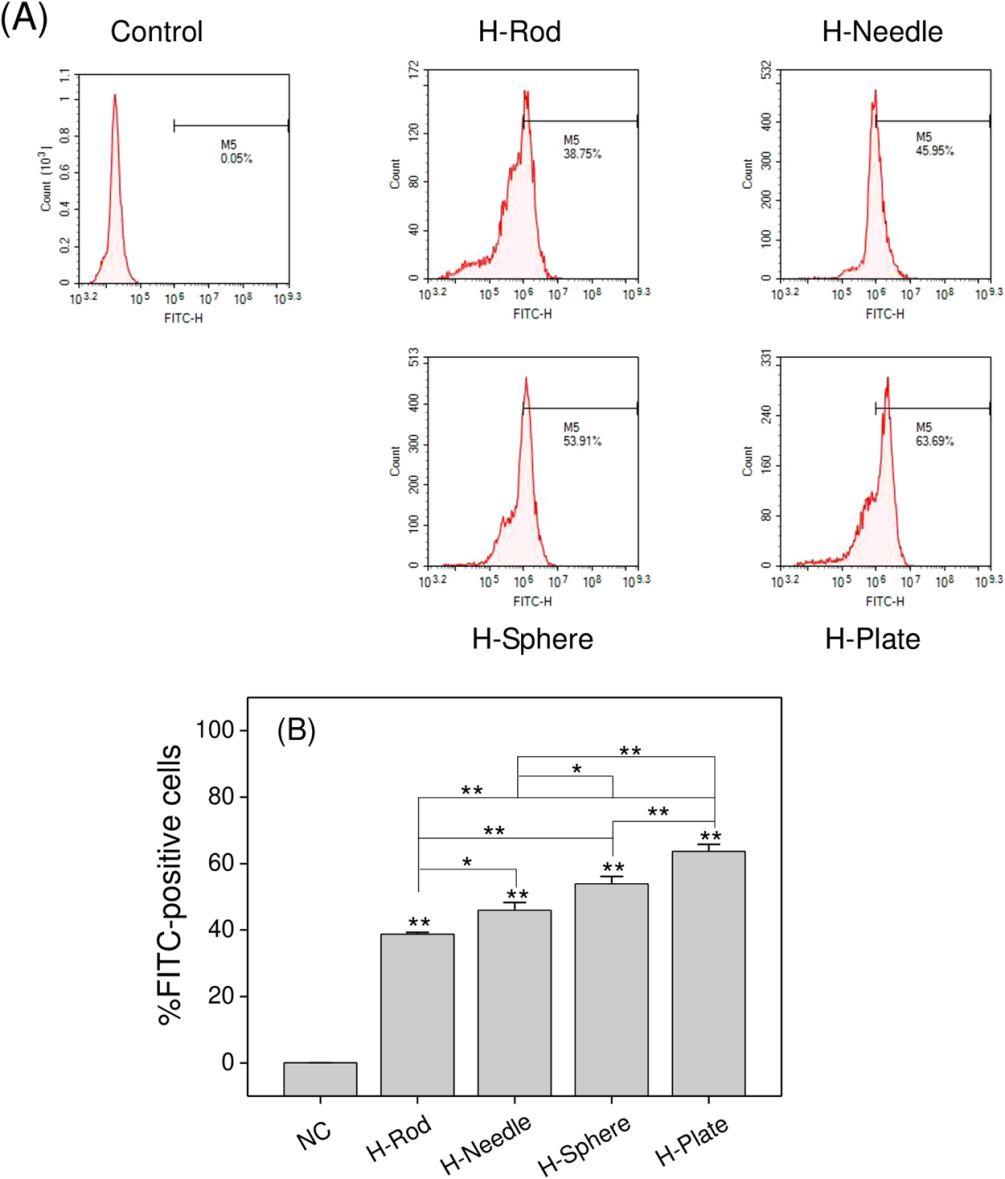
Flow cytometry analysis of internalized HAP crystals (200 μg/mL) with various shapes. (**A**) Flow cytometric data of endocytosis. (**B**) Quantitative results of endocytosis. The values represent mean ± SD (n = 3), *P < 0.05, **P < 0.01, compared with the control group.

After the A7R5 cells were exposed to the FITC-labeled HAP crystals with varying shapes for 6 h, the bound HAP was removed by using 0.4 mL of EDTA^19^. The proportion of the cells with endocytic crystals was examined through flow cytometry (Fig. 11A). The cells with positive FITC signaling can be considered as cells with endocytic crystals. All of the four types of HAP crystals could be endocytosed by A7R5 cells. The percentage of the cells with endocytic crystals was ranked in the following order: H-Plate > H-Sphere > H-Needle > H-Rod (Fig. 11B).

The cells with endocytic crystals for H-Plate was greater than that of H-Sphere probably because flat nanoparticles easily penetrate the lipid bilayer of the cell membrane and successfully enter cells; conversely, spherical nanoparticles tend to affect the membrane, prefer to stay near the membrane center, and stay on the membrane for a long time before they enter cells^36^.

### Changes in intracellular Ca^2+^ in A7R5 cells treated with HAP with various shapes

Excessive generated Ca^2+^ can cause cell damage and even cell death^37^. Fluo-4/AM is a fluorescent dye that can penetrate cell membranes. Fluo-4/AM that successfully enters a cell can be cleaved by intracellular esterase to form Fluo-4. Fluo-4 can combine with Ca^2+^ to produce strong green fluorescence. Thus, the concentration of intracellular Ca^2+^ can be determined by detecting the percentage of Furo-4 positive cells^38^.

**Figure 12:**
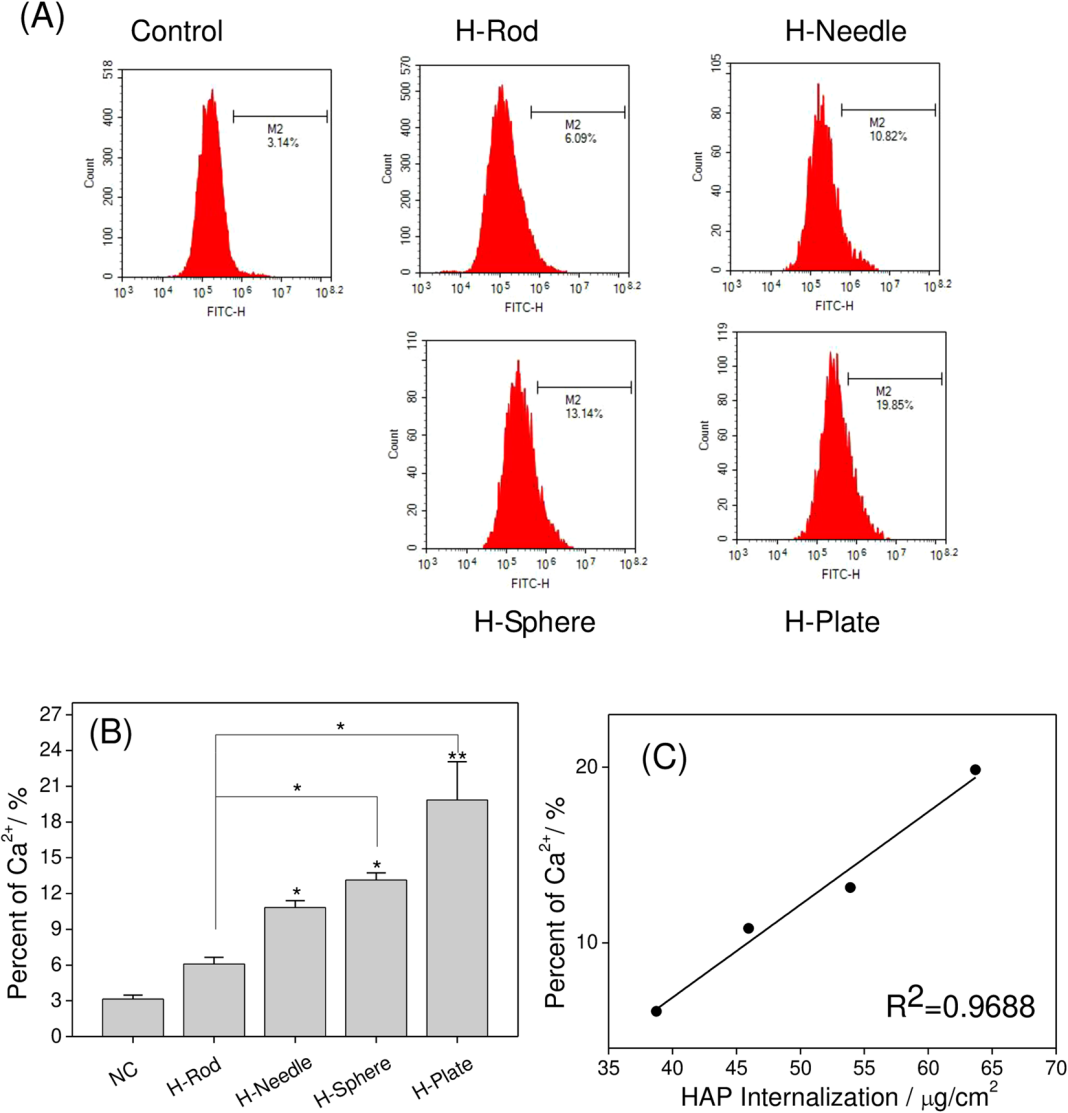
Changes of intracellular calcium concentration in A7R5 cells after exposure to 200 μg/mL HAP crystals with various shapes for 24 h. (**A**) Flow cytometric data of intracellular calcium elevation; (**B**) Quantitative results of intracellular calcium elevation. (**C**) Correlation curve between endocytosis and intracellular calcium concentration. The values represent mean ± SD (n = 3), *P < 0.05, **P < 0.01, compared with the control group.

In Fig. 12, the four types of HAP caused an increase in intracellular Ca^2+^ compared with that in the normal group. The intracellular Ca^2+^ fluorescence ratios in the control group, H-Rod, H-Needle, H-Sphere, and H-Plate were 3.14%, 6.09%, 10.82%, 13.14%, and 19.85%, respectively.

Figure 12C shows the correlation analysis result. HAP endocytosis by the cells was positively correlated with intracellular Ca^2+^ (R^2^ = 0.9688). A high degree of crystal endocytosis corresponded to a high intracellular calcium concentration.

### Changes in ALP activity in A7R5 cells treated with HAP with various shapes

**Figure 13:**
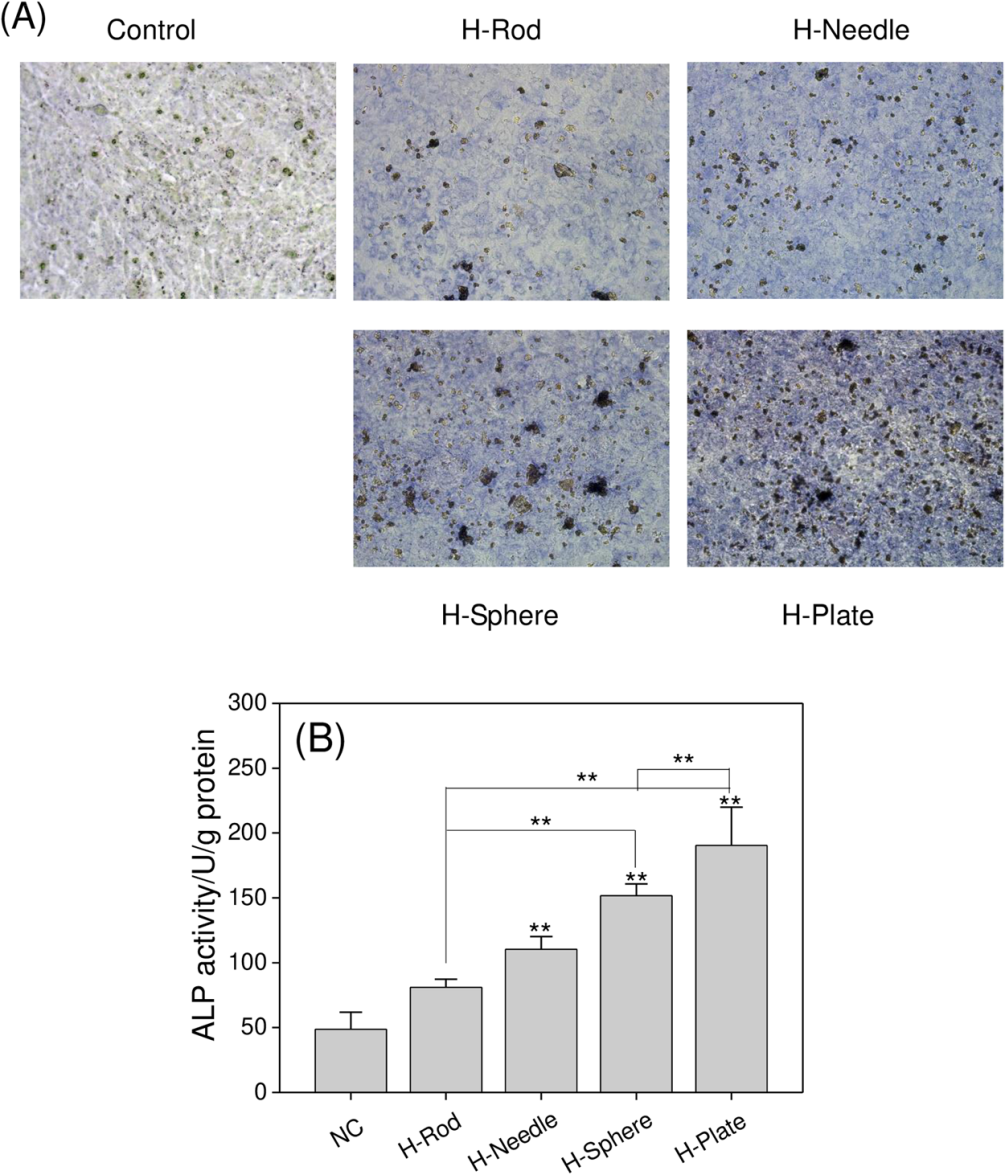
ALP activity of A7R5 cell after interaction with 200 μg/mL HAP crystals with various shapes for 14 d. (**A**) Microscope observation. (**B**) Quantitative results of ALP activity. The values represent mean ± SD (n = 5), **P < 0.01, compared with the control group.

ALP is an early marker of osteoblast formation^10^. The ALP expression in normal VSMCs is low, and its expression levels in calcified blood vessels and heart valves are obviously high. The four HAP nanoparticles promoted the activity of ALP (Fig. 13), indicating that HAP promoted mineralization. The levels of ALP expression induced by H-Rod, H-Needle, H-Sphere, and H-Plate increased by 32.32, 61.67, 103, and 141.62 U/g compared with those of the normal group, respectively.

### High expression of BMP-2, Runx2 and OCN in A7R5 cells

BMP-2 is one of the most important extracellular signaling molecules that promote bone formation and induce osteoblast differentiation. As a target gene of BMP-2, Runx2 is also an important regulator of osteoblast differentiation and bone development, and OCN is regarded as an osteogenesis marker^10^.

**Figure 14:**
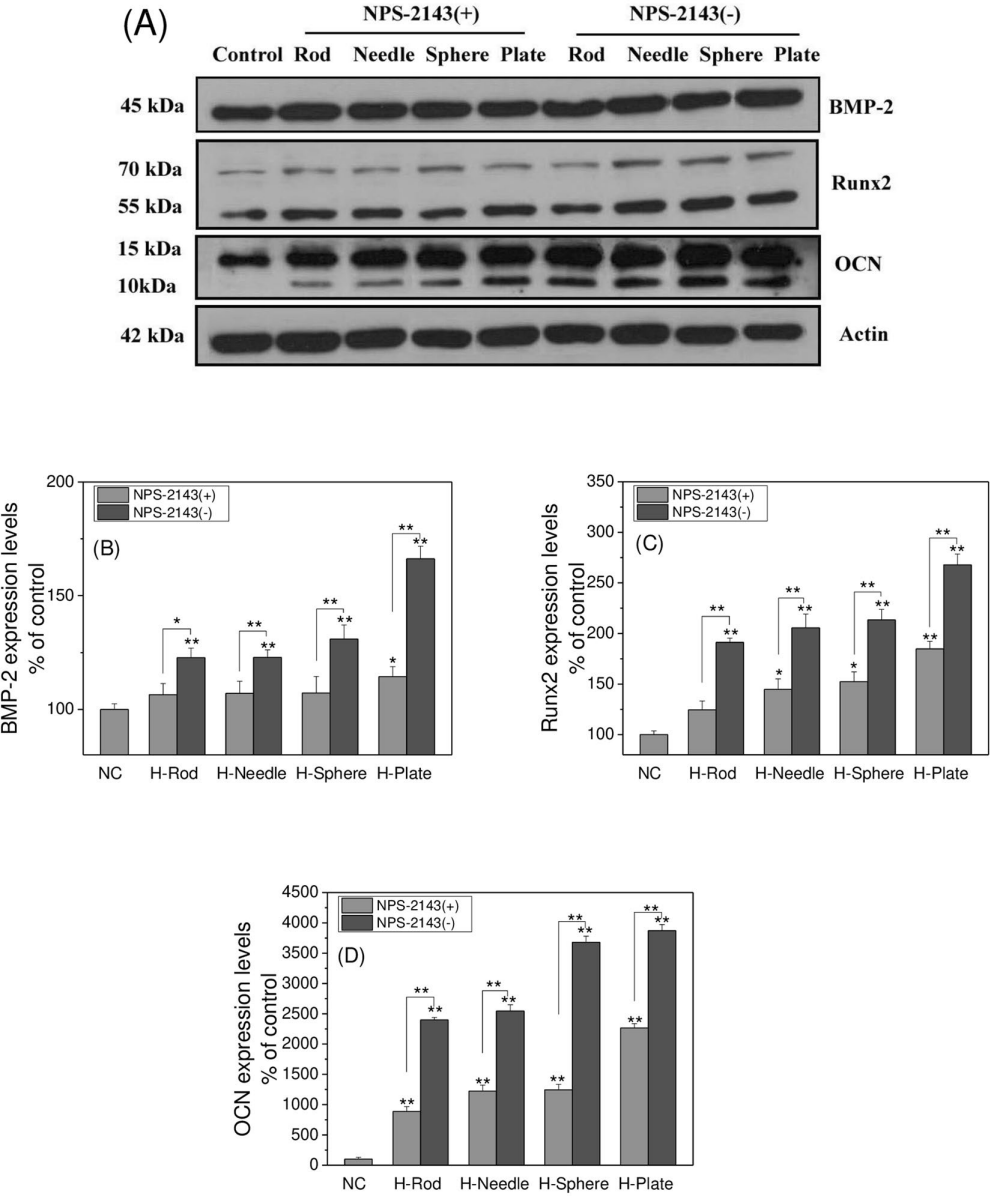
The osteogenic protein (BMP-2, Runx2 and OCN) expression in A7R5 cell after interaction with 200 μg/mL HAP crystals with various shapes for 14 d. Western-blot analysis of BMP-2, Runx2 and OCN (**A**); The relative protein quantitation of BMP-2 (**B**), Runx2 (**C**) and OCN (**D**). The values represent mean ± SD (n = 3), *P < 0.05, **P < 0.01, compared with the control group.

NPS-2134 is a common Ca-sensing receptor inhibitor. NPS-2143 can block the expression of CaSR, which can further affect the expression of BMP-2 and Runx2. The protein expression levels of BMP-2, Runx2, and OCN were assayed via Western blot analysis. As shown in Fig. 14, after 14 days of treatment with different morphological HAP groups, the protein levels of BMP-2, Runx2, and OCN significantly increased, and the osteogenic transformation induced by HAP-Plate and HAP-Sphere in A7R5 cells was more severe than that caused by HAP-Rod and HAP-Needle (Fig. 14B–D).

Figure 14 also shows that the differentiation of A7R5 cells to osteoblast-like cells under calcification conditions was mediated by CaSR-stimulated BMP-2 and Runx2 signaling pathways. Under the action of BMP-2 and Runx2 osteogenic factors, cells expressed excessive osteogenic proteins (OCN and ALP), induced osteogenic transformations, and increased the risks of vascular calcification.

## Discussion

### Toxicity mechanism of HAP with various shapes on A7R5 cells

Extracellular LDH levels are important indicators to verify changes in cell membrane permeability^39^. Adherent HAP crystals caused the release of LDH (Fig. 3B), demonstrating that HAP caused an increase in cell membrane permeability. Cell membrane rupture can cause intracellular electrolyte disorders, produce large amounts of ROS (Fig. 5). Excessive ROS generation by exogenous particles can induce oxidative stress^40^, which is a vital mechanism of cell toxicity^41^. Excessive ROS formation can also induce a decrease in Δψm (Fig. 7)^42^. Decreased Δψm often precedes cellular apoptosis and necrosis^43^.

HAP crystals were endocytosed by A7R5 cells (Figs. 10 and 11), leading to cell membrane rupture (Fig. 3B). Endocytosis occurs after HAP crystals interact with cells for 1 h^44^. Therefore, HAP may damage the cell membrane after crystal endocytosis. And cell membrane damage also caused by the adhered HAP^45^. Extracellular particles can enter cells through macrophagocytosis and membrane rupture^46^.

Lysosomes contain many acidic hydrolyzing enzymes, and their pH is approximately 4.5^47^. In our study, the HAP crystals were dissolved via the acid hydrolyzing enzymes, thereby causing a remarkable increase in intracellular Ca^2+^ (Fig. 12), which destroyed the osmotic pressure balance on lysosomal membranes, causing excessive lysosomal disruption and cell necrosis (Fig. 8)^48^.

The intracellular Ca^2+^ production caused by HAP with various shapes differed. High amounts of H-Plate and H-Sphere released high Ca^2+^ contents, resulting in high rates of cell necrosis (Fig. 8).

### Potential vascular calcification risk differences caused by HAP with different shapes

**Figure 15:**
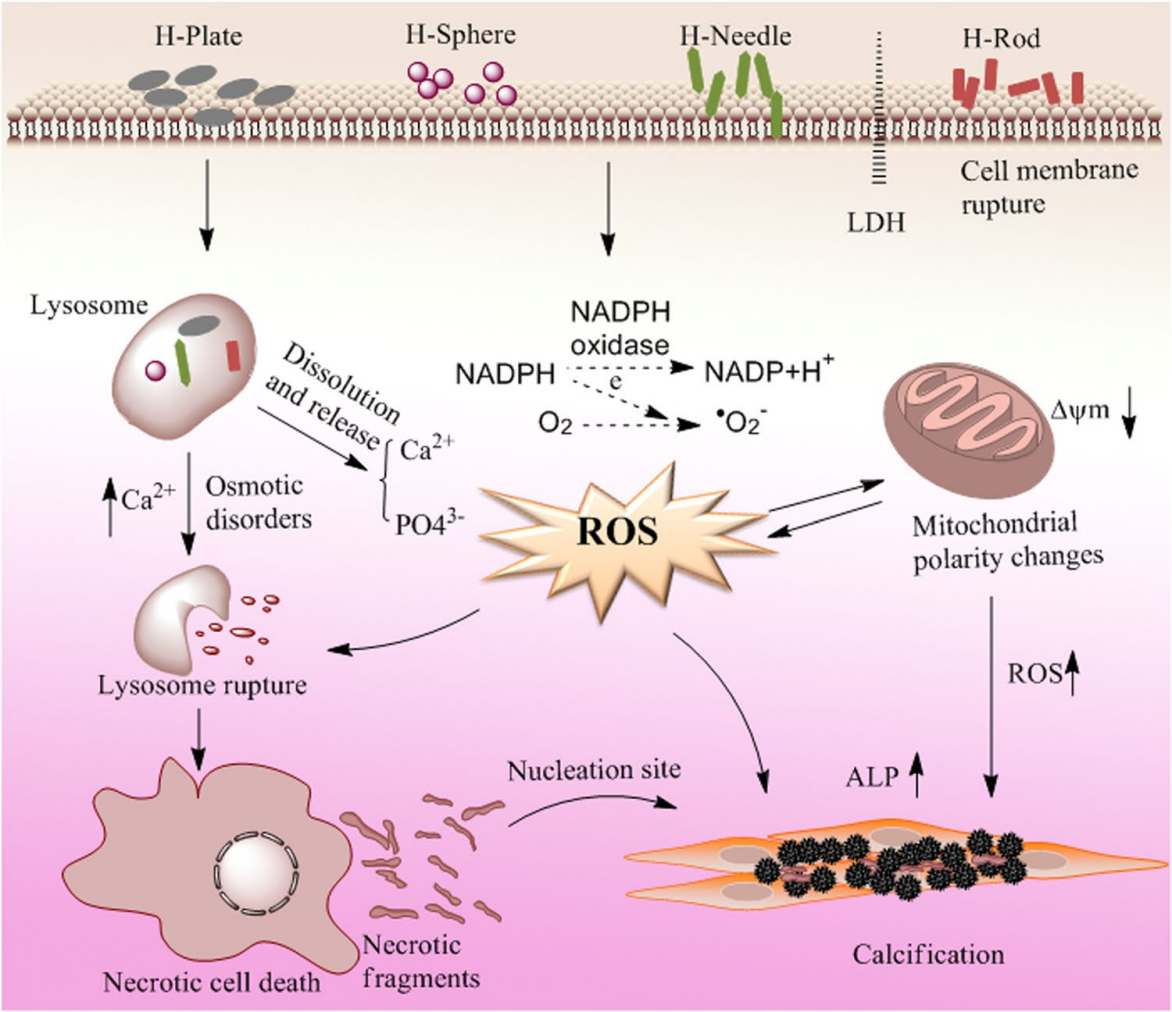
Schematic of the mechanism of cell injury and calcification caused by HAP nanoparticles with various shapes.

The mechanism of cell injury and calcification caused by HAP nanoparticles with various shapes is summarized in Fig. 15. Cell necrosis may be more likely to cause vascular calcification than apoptosis^49^. The increasing cell volume caused cell to rupture and produce a large number of membrane-like necrotic fragments, which promote the formation of HAP crystals. Thereby, H-Plate and H-Sphere generated more calcium deposits (Fig. 9). The increase in intracellular Ca^2+^ was also an important cause of the activation of HAP formation. Massive Ca^2+^ caused the depletion of calcium inhibitor and the exposure of the protein annexin AnxA6/phosphatidylserine to the matrix vesicle surface, which can provide nucleation sites for HAP and increase calcium deposition^50^. The deposited HAP on the cell surface and the endocytosed crystals in the cells led to excessive ROS production^51–53^, thereby increasing the ALP activity and eventually mediating the osteogenic differentiation. HAP promotes the expression of osteogenic protein, and BMP-2 and Runx2 have synergistic effects on osteoblast differentiation. Runx2 can promote the expression of ALP and OCN^54^. Therefore, the production of membrane fragments caused by cell necrosis, excessive ROS production, increase in intracellular Ca^2+^, and osteogenic protein expressions could promote calcium deposition and osteogenesis transformation of A7R5 cells. As a result, vascular calcification occurred.

Calcium deposition occurs in two ways: the continuous growth of adhered HAP crystals on the cell surface^55,56^ and the direct deposition of inorganic calcium and inorganic phosphorus components in the medium to the active sites on the damaged cell surface^57,58^. The culture medium contained 1.4 mmol/L inorganic phosphorus (KH_2_PO_4_) and 2 mmol/L inorganic calcium (CaCl_2_)^58^. However, their amounts were insufficient to form the amount of calcium phosphate deposition shown in Fig. 9. Therefore, the increase in calcium deposition was due to the continuous growth and deposition of inorganic calcium and phosphorus in the culture medium on the surface of the adhered HAP crystal.

### Factors affecting the cytotoxicity of HAP crystals

The cytotoxicity of HAP crystals with different morphologies is not determined by a single factor. Cytotoxicity is affected by various physical parameters, including S_BET_, electrical conductivity, and Zeta of crystals. In addition, the cytotoxic effect of HAP is affected by cell membrane interaction, endocytosis, and intracellular Ca^2+^ release.

1) The HAP toxicity was affected by S_BET_, conductivity, and Zeta of crystal

- Influence of the S_BET_ of HAP
  Particles with large specific surface area are more likely to generate ROS^41,59^. Therefore, H-Plate and H-Sphere had a larger S_BET_ and a stronger ability to generate ROS, thus causing serious cell damage.
  The greater the crystal adhesion is, the greater the cell damage will be^9,60^. H-Plate crystal had the largest S_BET_, the largest crystal plane exposure, and the largest contact area, therefore, this crystal had the strongest interaction and the most serious damage to cells.
- b) Effect of HAP conductivity
  When nanoparticles with a high conductivity interact with cells, they likely cause cell osmotic pressure imbalance and cell damage. In our study, the conductivities of H-Plate and H-Sphere were higher than those of H-Needle and H-Rod. Thus, H-Plate and H-Sphere had greater damage to cells.
- c) Effect of the zeta potential of HAP
  The zeta potential values of HAP in pure water and culture medium were negative because of the abundance of the anionic P–OH group on the crystal surface^61^. Wilhelm *et al*.^62^ suggested that the adsorption of negatively charged particles at positively charged sites via electrostatic interaction can lead to localized neutralization and subsequent bending of the membrane, thereby causing cellular uptake. In usual cases, the cell surface is weakly electronegative^63^. The toxicities of H-Sphere, H-Needle, and H-Rod crystals decreased as their negative zeta potential charge increased. The absolute zeta value of the H-Sphere crystal in the culture medium was the smallest, therefore, the highest affinity was observed in A7R5 cells, resulting in high cytotoxicity. H-Plate crystals had more negative charges, but their cytotoxicity was the greatest, which possibly because of their maximum S_BET_ and high degree of endocytosis.
  2) HAP toxicity was affected by cell membrane interaction, endocytosis, and intracellular Ca^2+^ release.
- a) The adhered HAP crystals would induce the breakdown of membrane lipids and the release of LDH (Fig. 3B), leading to cell membrane rupture. Severe cell membrane rupture would cause necrotic cell death (Fig. 8).
- b) Nanocrystals were endocytosed by cells (Fig. 10). The endocytosed nanocrystals induced a decrease in Δψm (Fig. 7), destroyed lysosomal integrity (Fig. 6), and caused cell necrosis (Fig. 8).
- c) The endocytosed HAP crystals caused a remarkable increase in intracellular Ca^2+^ (Fig. 12). The sudden and intense release of Ca^2+^ destroyed the osmotic pressure balance on lysosomal membranes^48^, causing excessive lysosomal disruption and cell necrosis (Fig. 8).

## Conclusions

Four kinds of HAP nanoparticles with various shapes damaged the A7R5 cells to different degrees, resulting in decreased cell viability, disorganized cell morphology, disrupted cell membranes, increased intracellular ROS generation, decreased Δψm, decreased lysosome integrity, increased ALP, and increased intracellular calcium concentration, thereby leading to cell necrosis. The HAP-induced cytotoxicity showed the following trend: H-Plate > H-Sphere > H-Needle > H-Rod. The nano-HAP with a large S_BET_, a high electrical conductivity, and a low Zeta elicited high cytotoxic effects. More calcium deposits on the cell surface, higher expression levels of osteogenic protein (BMP-2, Runx2, OCN, and ALP), and a stronger osteogenic transformation ability were observed in the crystal with a high cell cytotoxicity. This study could provide insights into the mechanism on how nano-HAPs injured vascular smooth muscle cells and induced vascular calcification.
